# Statin use and Vital Organ Failure in Patients With Asthma–Chronic Obstructive Pulmonary Disease Overlap: A Time-Dependent Population-Based Study

**DOI:** 10.3389/fphar.2019.00889

**Published:** 2019-08-16

**Authors:** Jun-Jun Yeh, Shih-Huei Syue, Cheng-Li Lin, Chung Y. Hsu, Zonyin Shae, Chia-Hung Kao

**Affiliations:** ^1^Department of Family Medicine, Ditmanson Medical Foundation Chia-Yi Christian Hospital, Chiayi, Taiwan; ^2^Department of Childhood Education and Nursery, Chia Nan University of Pharmacy and Science, Tainan, Taiwan; ^3^College of Medicine, China Medical University, Taichung, Taiwan; ^4^Department of Nursing, Mei-Ho University, Pingtung, Taiwan; ^5^Management Office for Health Data, China Medical University Hospital, Taichung, Taiwan; ^6^College of Medicine, China Medical University, Taichung, Taiwan; ^7^Graduate Institute of Biomedical Sciences and School of Medicine, College of Medicine, China Medical University, Taichung, Taiwan; ^8^Department of Computer Science and Information Engineering, Asia University, Taichung, Taiwan; ^9^Department of Nuclear Medicine and PET Center, China Medical University Hospital, Taichung, Taiwan; ^10^Department of Bioinformatics and Medical Engineering, Asia University, Taichung, Taiwan

**Keywords:** asthma–chronic obstructive pulmonary disease overlap (ACO), hepatic failure, renal failure, heart failure, respiratory failure

## Abstract

**Objective:** The effects of statins on the risk of hepatic, renal, respiratory, and heart failure among patients with asthma–chronic obstructive pulmonary disease overlap (ACO) have not been reported.

**Design:** Time-dependent population-based study.

**Setting:** Patient data from 2000 to 2010 were retrieved from the Taiwan National Health Insurance Research Database.

**Patients:** We divided patients with ACO into cohorts of statin use (N = 1,211) and nonuse (N = 7,443).

**Measurements and Main Results:** The cumulative incidence rates of hepatic, renal, respiratory, and heart failure were analyzed through Cox proportional regression analysis with time-dependent variables. After adjustment for multiple confounding factors, including age, sex, comorbidities, and medications [statins, inhaled corticosteroid (ICS), or oral steroid (OS)], the adjusted hazard ratios (aHRs) [95% confidence intervals (CIs)] for hepatic, renal, respiratory, and heart failure were 0.50 (0.40–0.64), 0.49 (0.38–0.64), 0.61 (0.27–2.21), and 0.47 (0.37–0.60), respectively. The aHRs (95% CIs) for statin use with [ICS, OS] for hepatic, renal, and heart failure were [0.36 (0.20–0.66), 0.52 (0.39–0.70)]; [0.82 (0.51–1.34), 0.46 (0.33–0.63)]; and [0.66 (0.40–1.07), 0.48 (0.37–0.64)], respectively.

**Conclusions:** The ACO cohort with statin use exhibited lower risk of hepatic, renal, and heart failure than any other cohort, regardless of age, sex, comorbidities, or ICS or OS use. Regarding the combined use of statins and ICS, the risks of hepatic failure were lower. For the combined use of statins and OS, hepatic, renal, and heart failure were less frequent.

## Introduction

Multiple organ failure is a serious concern in hospitals (Valley et al., 2015). It can include respiratory failure (hypoxemia), reduction in the ration of oxygen pressure in arterial blood (PaO2) to inspired oxygen fraction (FiO2), a requirement for mechanical ventilation, cardiac failure (hypotension unresponsive to adequate fluid resuscitation and requiring vasopressors), renal failure (diminished urine output), increased serum creatinine, and hepatic failure (bilirubin increased, liver function impaired, prothrombin time prolonged). Respiratory failure is usually followed by cardiac, renal, and hepatic failure (Valley et al., 2015). Multiple organ failure is a clinical syndrome characterized by the functional deterioration of two or more organs or organ systems. Evidence suggests that sepsis is the most frequent cause of organ function deterioration. The pathophysiology is the following: 1) Monocyte stimulation by exotoxins and endotoxins results in the release of proinflammatory cytokines [e.g., tumor necrosis factor alpha (TNF-α) and interleukin-6 (IL-6)], increased susceptibility of monocytes to aggregation on the endothelium, and enhanced release of other inflammatory and anti-inflammatory cytokines. 2) Stimulation of platelet aggregation and thrombin formation result in thrombosis in microvasculature and impaired circulation in individual organs or organ systems (Chaudhry et al., 2013). Therefore, this process causes a cascade of events in multiple organ failure, and multiple organ failure may be considered as systemic inflammation.

High-density lipoprotein cholesterol decreases and low-density lipoprotein cholesterol increases before heart failure, renal failure, and liver failure. This dyslipidemia was also found in chronic obstructive pulmonary disease (COPD) and asthma.

Statins are cholesterol-lowering drugs used for primary or secondary prevention of cardiovascular diseases (Afilalo et al., 2007). In addition to the primary effect, statins act beneficially through different pleiotropic mechanisms on inflammation, fibrosis, endothelial function, thrombosis, and coagulation to ameliorate chronic liver diseases (Jones, 2006). Statins also affect the airways (Wang et al., 2015) and pulmonary vessels (Zhang et al., 2017). However, this effect in multiple organ failure is speculative (Omar et al., 2017).

Asthma–COPD overlap (ACO) is a disorder combining the components of both COPD and asthma. A higher frequency of cardiovascular disease, stroke, pneumonia, pulmonary embolism, and tuberculosis was associated with acute exacerbation of COPD or asthma (Yeh et al., 2018). Consequently, the incidence of cardiac, hepatic, renal, and respiratory failure increased (Llanos et al., 2018). The roles of statins, inhaled corticosteroid (ICS), and oral steroid (OS) in these vital organ failures are unclear from the English literature. Therefore, we identified this topic based on the general population.

## Material and Methods

### Data Source

This retrospective cohort study based on the Longitudinal Health Insurance Database selected 1 million insurants from the Taiwan National Health Insurance (NHI) program from 2000 to 2011. Implemented in 1995, the NHI program supplies comprehensive medical care embodying ambulatory and inpatient care for almost all of Taiwan’s population (Database NHIR). Details of the NHI program are available in a previous study (Wang et al., 2018). Diseases were defined by the *International Classification of Diseases, Ninth Revision, Clinical Modification* (*ICD-9-CM*) codes. The Research Ethics Committee of China Medical University and Hospital in Taiwan approved the study (CMUH104-REC2-115-CR3).

### Sampled Participants

This retrospective cohort study analyzed the effects of statins, ICS, and OS exposure on patients with ACO. The prevalence of ACO among young adults was found in many studies in the real world (de Marco et al., 2013; de Marco et al., 2015; Ekerljung et al., 2018), especially in the aboriginal population (Koleade et al., 2018). We identified patients aged 20 years and above with ACO and diagnosed with COPD (*ICD-9-CM* codes 491, 492, and 496) from January 1, 2000, to December 31, 2010. The date of the first diagnosis of asthma (*ICD-9-CM* code 493) was selected as the index date. These patients had experienced at least one COPD or asthma-related examination such as a pulmonary function test (PFT), immunoglobulin E (IGE), eosinophil count with chest X-ray (CXR), or chest computer tomography (CT). In Taiwan, the demographic characteristics reveal that 82.9% of COPD patients were smokers, 84.7% had abnormal CXR (e.g., emphysema or chronic bronchitis), and 58.7% had a PFT (Ho et al., 2018). The sensitivity of the diagnosis of asthma and COPD were high, up to 92.0% and 86.2%. Meanwhile, we used the ICS and OS use status of the patients for confirming asthma–chronic pulmonary disease overlap syndrome (ACOS). Thus, deriving the ACO from the COPD cohort in the National Health Insurance Research Database (NHIRD) is reasonable (Yeh et al., 2019). Patients with a record of multiple vital organ failure including hepatic failure (*ICD-9-CM* codes 570, 571, and 572), renal failure (*ICD-9-CM* codes 584, 585, 586, 403.01, 403.11, 403.91, 404.02, 404.03, 404.12, 404.13, 404.92, 404.93, 582, 588, V42.0, V45.1, V56.2, 570, 571, and 572), respiratory failure (*ICD-9-CM* codes 5181, 5182, 5183, and 5184), or heart failure (*ICD-9-CM* code 428) prior to the asthma diagnosis were excluded. Each patient was followed up from the initial asthma diagnosis date until one of the following: the patient was removed from health insurance; hepatic, renal, respiratory, and heart failure occurred; or December 31, 2011. We were primarily interested in the distinction between ACO patients with and without statin exposure. Because the medical follow-up period was dynamic, we computed medicine exposure every 6 months (Zhang et al., 2017; Yeh et al., 2019). Patients who received the medicine at least 30 days after the date of ACO diagnosis were enrolled, and these patients received the statin before multiple vital organ failure (Zhang et al., 2017; Yeh et al., 2019).

Considering the influence of comorbidity and medication, we defined a person as having a certain comorbidity if the disease had been confirmed by an end point of multiple vital organ failure such as hepatic, renal, respiratory, and heart failure. Comorbidities consisted of sleep disorder (*ICD-9-CM* 780.50), diabetes (*ICD-9-CM* 250), hypertension (*ICD-9-CM* 401–405), hyperlipidemia (*ICD-9-CM* 272.0 272.4), coronary artery disease (CAD) (*ICD-9-CM* 410–414), stroke (*ICD-9-CM* 430–438), hepatitis B (*ICD-9-CM* 070.20, 070.22, 070.30, 070.32), and hepatitis C (*ICD-9-CM* 070.40, 070.44, 070.51, 070.54, 070.70, 070.71). Medications were ICS and OS. Patients who received ICS or OS over 30 days from January 2000 to December 2010 were included in this study (Ko et al., 2019). The statin cohort and nonstatin cohort were matched at a 1:1 ratio based on propensity scores (Byrne et al., 2019; Ko et al., 2019) (**Supplement Table 1**).

The World Health Organization (WHO) recommends the use of the defined daily dose (DDD) and prescribed daily dose (PDD) methodology in drug utilization studies. The definition of the DDD is the assumed average maintenance dose per day for a drug used for its main indication in adults. DDDs are assigned by the WHO. For instance, the DDD for atorvastatin is 20 mg. The PDD is defined as the average dose prescribed according to a representative sample of prescriptions. The DDDs for statins remained unchanged from 1997. However, in January 2009, alterations were made to the DDD for five of the six statins in order to better reflect the current daily dosages. The DDDs for atorvastatin and simvastatin have been doubled, whereas for pravastatin, lovastatin, and fluvastatin the DDDs, have been increased by 50% (Godman et al., 2014). There are different DDDs in different stages during this study. To avoid this dosage bias, we used the length of use in days (>30 days) for the analysis (Pedan et al., 2007; Ko et al., 2019).

### Statistical Analysis

Student’s t-test was used for calculating the mean age, and the chi-square test was used for age, sex, strata of comorbidity, and strata of medication for the statin use and nonuse cohorts. The Kaplan–Meier method was applied to derive the cumulative incidences of hepatic failure, renal failure, and heart failure between statin users and nonusers and to detect differences through log-rank testing.

We defined the incidence rate (IR) as the number of events divided by the total person-years. Because those ACO patients taking statins fluctuated in terms of follow-up, we set statin as a time-dependent covariate in the Cox proportional hazards model to demonstrate the effects as hazard ratios (HRs) and corresponding 95% confidence intervals (CIs). The time-dependent covariate of statins indicated that intrapatient comparison may be performed. The drug exposure variables were quantified as a binary (yes/no) variable every 6 months. The adjusted HRs were estimated after controlling for age, sex, comorbidity, and medication. Furthermore, we dissected the IRs and HRs of multiple vital organ failure including hepatic failure, renal failure, and heart failure according to sex, age, ICS status, and OS status for statin users and nonusers. Analyses were conducted and data collected using SAS 9.4 software (SAS Institute, Cary, NC, USA). Statistical significance was set at P < 0.05.

### Results

Among 8,654 patients with ACO, 1,211 had been exposed to statins. **Table 1** indicates that the mean age of statin users was less than that of nonusers (62.9 vs. 64.1 years, P = 0.001). The distributions of statin users and nonusers differed according to age, sex, strata of comorbidity (except hepatitis B and C), and strata of medication [age, P < 0.001; sex, P < 0.001; strata of comorbidity (except hepatitis B and C), all P < 0.001; strata of medication: ICS, P = 0.02; OS, P < 0.001].

**Table 1:** Distribution of demographic and clinical comorbidity data in study cohorts.

Variables	ACO						p-value
	Statin						
	All (N = 8,654)		No (N = 7,443)		Yes (N = 1,211)		
	n	%	n	%	n	%	
**Age, years**							<0.001***
<50	1,599	(18.5)	1,418	(19.1)	181	(15.0)	
50–64	2,418	(27.9)	1,962	(26.4)	456	(37.7)	
65+	4,637	(53.6)	4,063	(54.6)	574	(47.4)	
Mean (SD)^a^	64.0	14.8	64.1	15.3	62.9	11.9	0.001**
**Gender**							<0.001***
Women	3,722	43.0	3,090	41.5	632	52.2	
Men	4,932	57.0	4,353	58.5	579	47.8	
**Comorbidity**							
Sleep disorder	3,578	41.4	2,962	39.8	616	50.9	<0.001***
Diabetes	1,455	16.8	1,093	14.7	362	29.9	<0.001***
Hypertension	5,628	65.0	4,648	62.5	980	80.9	<0.001***
Hyperlipidemia	2,825	32.6	1,898	25.5	927	76.6	<0.001***
CAD	3,576	41.3	2,917	39.2	659	54.4	<0.001***
Stroke	1,415	16.4	1,205	16.2	210	17.3	<0.001***
Hepatitis B	326	3.77	281	3.78	45	3.72	0.92
Hepatitis C	135	1.56	116	1.56	19	1.57	0.98
**Medication**							
Inhaled corticosteroids (ICSs)	2,064	23.9	1,743	23.4	321	26.5	0.02*
Oral steroids (OSs)	6,319	73.0	5,377	72.2	942	77.8	<0.001***

Chi-square test, ^a^ t-test.

*P < 0.05, **P < 0.01, ***P < 0.001.

**Table 2** demonstrates that statin users with multiple vital organ failure were respectively 0.58, 0.68, and 0.44 times less likely to experience hepatic, renal, and heart failure than were nonusers (IR per 1,000 person-years: hepatic failure, 8.61 vs. 16.2; renal failure, 7.64 vs. 11.3; heart failure, 7.99 vs. 19.3), with adjusted HRs of 0.5, 0.49, and 0.47 (hepatic failure, 95% CI = 0.40–0.64; renal failure, 95% CI = 0.38–0.64; heart failure, 95% CI = 0.37–0.60). Comparing statin use with nonuse, the adjusted HR for respiratory failure for statin use was 0.61 (0.17–2.21).

**Table 2:** Overall incidence of hepatic failure, renal failure, respiratory failure, and heart failure (per 1,000 person-years) and estimated HRs in ACO patients taking statins compared with ACO patients without statins using a time-dependent regression model.

	Statin	
Variables	No (N = 7,443)	Yes (N = 1,211)
**Hepatic failure**		
Person-years	42822	9519
Follow-up time (y), mean ± SD	5.75 ± 3.45	7.86 ± 2.94
Event, n	694	82
Rate	16.2	8.61
cHR (95% CI)	1 (reference)	0.58 (0.46, 0.72)***
aHR (95% CI)^a^	1 (reference)	0.50 (0.40, 0.64)***
**Renal failure**		
Person-years	45,034	9,684
Follow-up time (y), mean ± SD	6.05 ± 3.45	8.00 ± 3.41
Event, n	510	74
Rate	11.3	7.64
cHR (95% CI)	1 (reference)	0.68 (0.53, 0.87)**
aHR (95% CI)^a^	1 (reference)	0.49 (0.38, 0.64)***
**Respiratory failure**		
Person-years	46,898	9,897
Follow-up time (y), mean ± SD	6.30 ± 3.42	8.17 ± 2.78
Event, n	20	3
Rate	0.43	0.30
cHR (95% CI)	1 (reference)	0.68 (0.20, 2.30)
aHR (95% CI)^a^	1 (reference)	0.61 (0.17, 2.21)
**Heart failure**		
Person-years	43,215	9,461
Follow-up time (y), mean±SD	5.81 ± 3.50	7.96 ± 2.88
Event, n	835	77
Rate	19.3	7.99
cHR (95% CI)	1 (reference)	0.44 (0.35, 0.56)***
aHR (95% CI)^a^	1 (reference)	0.47 (0.37, 0.60)***

a Adjusted for age; sex; comorbidity of sleep disorder, diabetes, hypertension, hyperlipidemia, CAD, stroke, hepatitis B, and hepatitis C; and ICS and OS.

cHR, crude hazard ratio; aHR, adjusted hazard ratio; ICS, inhaled corticosteroid; OS, oral steroid.

**P < 0.01, ***P < 0.001.

**Table 3** reveals that women who took statins with multiple vital organ failure were respectively 0.57, 0.66, and 0.36 times less likely to experience hepatic, renal, and heart failure than women who did not (IR: hepatic failure, 7.88 vs. 15.0; renal failure, 6.00 vs. 8.96; heart failure, 5.81 vs. 17.5), with adjusted HRs of 0.47, 0.48, and 0.40 (hepatic failure, 95% CI = 0.33–0.67; renal failure, 95% CI = 0.33–0.72; heart failure, 95% CI = 0.27–0.58). Men who took statins were respectively 0.59 and 0.54 times less likely to experience hepatic and heart failure than men who did not (IR: hepatic failure, 9.45 vs. 17.1; heart failure, 10.5 vs. 20.8), with adjusted HRs of 0.54 and 0.55 (hepatic failure, 95% CI = 0.38–0.75; heart failure, 95% CI = 0.40–0.75). Men who took statins were half as likely to experience renal failure as were men who did not after adjustment for age, comorbidity, and medication (95% CI = 0.36–0.70). Statin users aged ≥50 years were respectively 0.58, 0.62, and 0.40 times as likely to experience hepatic, renal, and heart failure as nonusers aged ≥50 years (IR: hepatic failure, 8.78 vs. 16.5; renal failure, 8.50 vs. 13.8; heart failure, 9.18 vs. 24.7), with adjusted HRs of 0.53, 0.50, and 0.47 (hepatic failure, 95% CI = 0.40–0.68; renal failure, 95% CI = 0.38–0.65; heart failure, 95% CI = 0.37–0.60). Among patients aged <50 years, statin users were 0.55 times as likely to experience hepatic failure as nonusers (IR: hepatic failure, 7.76 vs. 15.2), with an adjusted HR of 0.29 (95% CI = 0.15–0.54). Among patients aged <50 years, statin users were 0.27 times as likely to experience renal failure as nonusers after sex, comorbidity, and medication were held constant (95% CI = 0.10–0.74).

**Table 3:** Overall incidence (per 1,000 person-years) and hazard ratio for hepatic failure, renal failure, and heart failure stratified by sex and age using a time-dependent regression model.

Variables	Men		Women	
	Statin		Statin	
	No (N = 4,353)	Yes (N = 579)	No (N = 3,090)	Yes (N = 632)
	Age < 50		Age ≧ 50	
	Statin		Statin	
	No (N = 1,418)	Yes (N = 181)	No (N = 6,025)	Yes (N = 1,030)
**Hepatic failure**				
No. of events	415	42	279	40
Incidence rate	17.1	9.45	15.0	7.88
cHR (95% CI)	1 (Reference)	0.59 (0.43, 0.81)**	1 (Reference)	0.57 (0.41, 0.80)**
aHR (95% CI)^a^	1 (Reference)	0.54 (0.38, 0.75)***	1 (Reference)	0.47 (0.33, 0.67)***
**Renal failure**				
No. of events	334	43	176	31
Incidence rate	13.2	9.52	8.96	6.00
cHR (95% CI)	1 (reference)	0.74 (0.54, 1.01)	1 (reference)	0.66 (0.45, 0.97)*
aHR (95% CI)^a^	1 (reference)	0.50 (0.36, 0.70)***	1 (reference)	0.48 (0.33, 0.72)***
**Heart failure**				
No. of events	508	47	327	30
Incidence rate	20.8	10.5	17.5	5.81
cHR (95% CI)	1 (Reference)	0.54 (0.40, 0.72)***	1 (reference)	0.36 (0.25, 0.52)***
aHR (95% CI)^a^	1 (Reference)	0.55 (0.40, 0.75)***	1 (reference)	0.40 (0.27, 0.58)***
**Hepatic failure**				
No. of events	145	12	549	70
Incidence rate	15.2	7.76	16.5	8.78
cHR (95% CI)	1 (Reference)	0.55 (0.30, 0.99)*	1 (Reference)	0.58 (0.45, 0.74)***
aHR (95% CI)^a^	1 (Reference)	0.29 (0.15, 0.54)***	1 (Reference)	0.53 (0.40, 0.68)***
**Renal failure**				
No. of events	33	5	477	69
Incidence rate	3.19	3.19	13.8	8.50
cHR (95% CI)	1 (Reference)	1.02 (0.40, 2.62)	1 (Reference)	0.62 (0.48, 0.80)***
aHR (95% CI)^a^	1 (Reference)	0.27 (0.10, 0.74)*	1 (Reference)	0.50 (0.38, 0.65)***
**Heart failure**				
No. of events	25	3	810	74
Incidence rate	2.41	1.90	24.7	9.18
cHR (95% CI)	1 (Reference)	0.79 (0.24, 2.60)	1 (Reference)	0.40 (0.32, 0.51)***
aHR (95% CI)^a^	1 (Reference)	0.35 (0.10, 1.26)	1 (Reference)	0.47 (0.37, 0.60)***

a Adjusted for comorbidity of sleep disorder, diabetes, hypertension, hyperlipidemia, CAD, stroke, hepatitis B, and hepatitis C as well as ICS and OS use.

HD, cHR, crude hazard ratio; aHR, adjusted hazard ratio; ICS, inhaled corticosteroid; OS, oral steroid.

*P < 0.05, **P < 0.01, ***P < 0.001.

**Table 4** indicates that among takers of ICS, those who also took statins were 0.36 times as likely to experience hepatic failure as those who did not, after age, sex, and comorbidity were controlled (95% CI = 0.20–0.66). Likewise, among those who did not take ICS, statin users were respectively 0.59, 0.54, and 0.40 times as likely to experience hepatic, renal, and heart failure as nonusers (IR: hepatic failure, 10.0 vs. 18.8; renal failure, 6.79 vs. 12.9; heart failure, 7.97 vs. 21.7), with adjusted HRs of 0.53, 0.40, and 0.43 (hepatic failure, 95% CI = 0.41–0.69; renal failure, 95% CI = 0.29–0.55; heart failure, 95% CI = 0.32–0.57). Among patients on OS, statin takers were respectively 0.65, 0.61, and 0.47 times as likely to experience hepatic, renal, and heart failure as nontakers (IR: hepatic failure, 8.01 vs. 13.1; renal failure, 6.15 vs. 10.1; heart failure, 7.91 vs. 17.6), with adjusted HRs of 0.52, 0.46, and 0.48 (hepatic failure, 95% CI = 0.39–0.70; renal failure, 95% CI = 0.33–0.63; heart failure, 95% CI = 0.37–0.64). Without OS, statin takers were respectively 0.48 and 0.38 times as likely to experience hepatic and heart failure as nontakers (IR: hepatic failure, 10.9 vs. 25.1; heart failure, 8.28 vs. 24.0), with adjusted HRs of 0.45 and 0.42 (hepatic failure, 95% CI = 0.29–0.71; heart failure, 95% CI = 0.25–0.70); additionally, they were 0.57 times as likely to experience renal failure as nonusers, after adjustment for age, sex, and comorbidity (95% CI = 0.37–0.88).

**Table 4:** Overall incidence (per 1,000 person-years) and hazard ratio for hepatic failure, renal failure, and heart failure by ICS and OS status using a time-dependent regression model.

Variables	With ICSs		Without ICSs	
	Statin		Statin	
	No (N = 1743)	Yes (N = 321)	No (N = 5,700)	Yes (N = 890)
	With OSs		Without OSs	
	Statin		Statin	
	No (N = 5377)	Yes (N = 942)	No (N = 2,066)	Yes (N = 269)
**Hepatic failure**				
No. of events	99	13	595	69
Incidence rate	8.81	4.95	18.8	10.0
cHR (95% CI)	1 (Reference)	0.57 (0.32, 1.02)	1 (Reference)	0.59 (0.46, 0.75)***
aHR (95% CI)^a^	1 (Reference)	0.36 (0.20, 0.66)***	1 (Reference)	0.53 (0.41, 0.69)***
**Renal failure**				
No. of events	79	26	431	48
Incidence rate	6.88	9.95	12.9	6.79
cHR (95% CI)	1 (eference)	1.38 (0.89, 2.15)	1 (Reference)	0.54 (0.40, 0.73)***
aHR (95% CI)^a^	1 (reference)	0.82 (0.51, 1.34)	1 (Reference)	0.40 (0.29, 0.55)***
**Heart failure**				
No. of events	140	21	695	56
Incidence rate	12.5	8.04	21.7	7.97
cHR (95% CI)	1 (Reference)	0.65 (0.41, 1.02)	1 (Reference)	0.40 (0.30, 0.52)***
aHR (95% CI)^a^	1 (Reference)	0.66 (0.40, 1.07)	1 (Reference)	0.43 (0.32, 0.57)***
**Hepatic failure**				
No. of events	414	60	280	22
Incidence rate	13.1	8.01	25.1	10.9
cHR (95% CI)	1 (Reference)	0.65 (0.49, 0.85)**	1 (Reference)	0.48 (0.31, 0.75)**
aHR (95% CI)^a^	1 (Reference)	0.52 (0.39, 0.70)***	1 (Reference)	0.45 (0.29, 0.71)***
**Renal failure**				
No. of events	332	47	178	27
Incidence rate	10.1	6.15	14.5	13.2
cHR (95% CI)	1 (Reference)	0.61 (0.45, 0.82)**	1 (Reference)	0.93 (0.62, 1.40)
aHR (95% CI)^a^	1 (Reference)	0.46 (0.33, 0.63)***	1 (Reference)	0.57 (0.37, 0.88)*
**Heart failure**				
No. of events	555	60	280	17
Incidence rate	17.6	7.91	24.0	8.28
cHR (95% CI)	1 (Reference)	0.47 (0.36, 0.62)***	1 (Reference)	0.38 (0.23, 0.61)***
aHR (95% CI)^a^	1 (Reference)	0.48 (0.37, 0.64)***	1 (Reference)	0.42 (0.25, 0.70)***

a Adjusted for age; sex; and comorbidity of sleep disorder, diabetes, hypertension, hyperlipidemia, CAD, stroke, hepatitis B, and hepatitis C.

vHD, cHR, crude hazard ratio; aHR, adjusted hazard ratio; ICS, inhaled corticosteroid; OS, oral steroid.

*P < 0.05, **P < 0.01, ***P < 0.001.

Propensity score matching for sensitive analysis is shown in **Supplement Table 1** and **Supplement Table 2** (Zhang et al., 2017; Ko et al., 2019). The incidences for hepatic failure in the statin cohort and the propensity score–matched nonstatin cohort were 8.77 and 17.3 per 1,000 person-years, respectively. Statin users had a 0.51-fold lower risk compared with propensity score–matched nonstatin patients (95% CI = 0.38–0.66). Statin users also had a 0.45-fold lower risk of renal failure compared with propensity score–matched nonstatin patients (95% CI = 0.34–0.61). Statin users also had a 0.46-fold lower risk of heart failure compared with propensity score–matched nonstatin patients (95% CI = 0.34–0.61).

**Figure 1:**
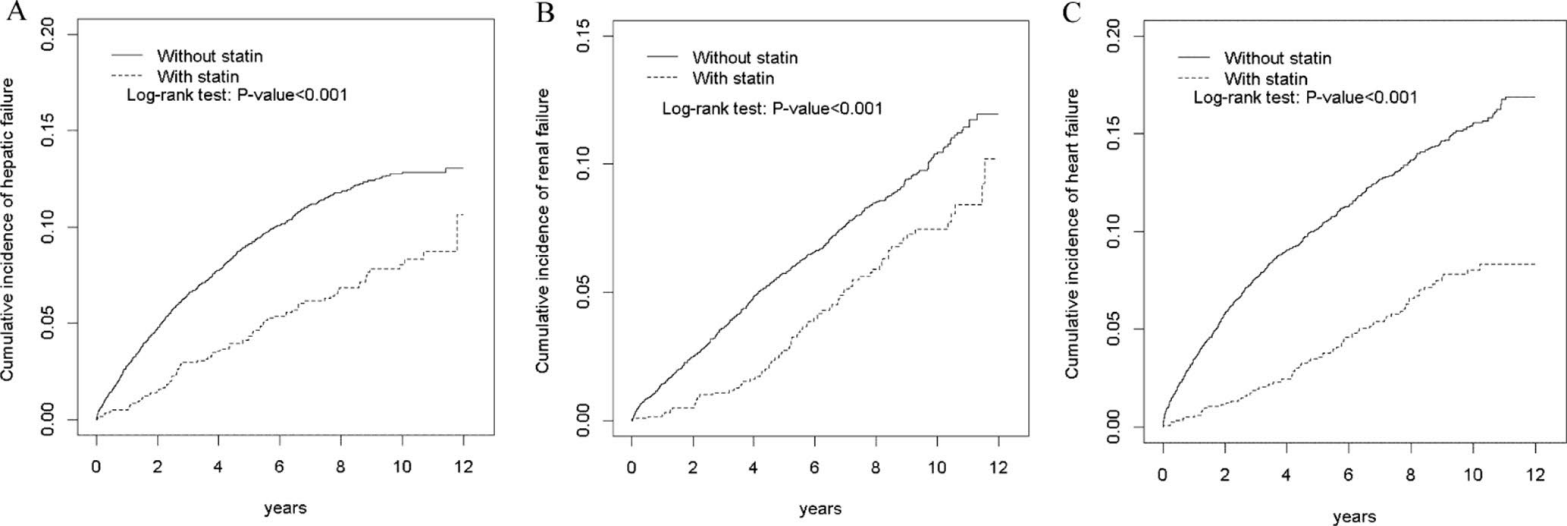
Cumulative incidence of hepatic failure **(A)**, renal failure **(B)**, and heart failure **(C)** compared between patients with and without statin use through the Kaplan–Meier method.

**Figure 1** illustrates that the cumulative incidences of statin users and nonusers differed significantly for hepatic, renal, and heart failure (log-rank test: hepatic failure, P < 0.001; renal failure, P < 0.001; heart failure, P < 0.001).

### Validation of the ACO Cohort

In their study based on the NHIRD in Taiwan, Su et al. reported that the frequency of ICS use among an ACOS cohort was 53.48% at follow-up. In addition, the ACO cohort of Shantakumar et al. (Shantakumar et al., 2018) received ICS in 46.1% of cases and OS in 85.5% of cases at 1-year follow-up. The validity of the *ICD-9-CM* codes for the diagnosis of COPD, as issued from an NHIRD report, was verified by physicians in 63.5% of patients. A high percentage (up to 58.7%) of patients received a PFT. Among the patients who received PFT, three were outpatients, and two were inpatients. In our study, the ACO cohort was derived from the COPD cohort (Ho et al., 2018). In addition, we identified the ACO cohort based on the *ICD-9-CM* code of COPD, asthma with ICS and OS (Cooke et al., 2011). The ACO cohort using statins had 26.5% on ICS and 77.8% on OS (Cooke et al., 2011). Physicians’ decision regarding ICS treatment should not only follow the treatment guidelines for the specific diseases but also observe the payment regulations stipulated by the NHI. These policies may prevent an indication bias.

### Validation of Hepatic, Renal, Heart, and Respiratory Failure

NHIRD data are de-identified and contain basic demographic information, disease diagnoses, prescriptions, operations, and investigations (Nan-Ping et al., 2013). While only a small number of validation studies with small sample sizes have been undertaken, they have generally reported positive predictive values of over 70% for various diagnoses (Lin et al., 2018). The catastrophic diseases included renal failure; respiratory failure and respiratory failure–related diseases pneumonia (sensitivity 94.7%) and tuberculosis (sensitivity 98.3%); heart failure–related disease (positive predictive value 96.6%) CAD (sensitivity 88.0%) (Cheng et al., 2014; Hsieh et al., 2019); and hepatic failure–related diseases liver tumor (sensitivity 91.5%) and cirrhosis (Hsieh et al., 2019). Issuance of catastrophic illness certificates was strict (Nan-Ping et al., 2013). Currently, patients cannot opt out of inclusion in the database, although this requirement is under review. In conclusion, the NHIRD is a large, powerful data source for biomedical research.

### Sensitivity Analysis and Healthy Use Bias

A higher health awareness and healthier lifestyle were found in patients using statins compared with nonusers (Shrank et al., 2011). Accordingly, statin users should be more likely to seek out preventive health services. In addition, the ACO cohort received more medical advice during regular follow-up. Moreover, we compared the ICS and OS effects on the risk of hepatic, renal, and heart failure in the ACO cohort between statin users and nonusers (Giovanni et al., 2014). We also stratified the ACO cohort into subgroups by sex, age (<50 or ≥50 years), IC use, and OS use for further sensitivity analysis. These sensitivity tests could help us to avoid healthy use bias. In addition, the frequency of the ACO cohort receiving CXR, PFT, sputum culture, and vaccinations was higher than the COPD cohort during the follow-up course. Meanwhile, regarding the severity of the late course of ACO with multiple vital organ failure (Chung et al., 2015; Shantakumar et al., 2018), a large portion, up to 98.7%, of patients with ACO (8554/8654) underwent eosinophil count, IGE, spirometry, and thoracic imaging as mentioned before (Yeh et al., 2019).

Measuring lifestyle factors, disease prevention behaviors, and drug compliance is difficult in observational studies. Sleep disorders are associated with lifestyle, income, and urbanization level (Cyril et al., 2013). To reduce the effect of confounding from healthy user bias, we used diseases such as sleep disorder, hypertension, and diabetes in individual insurance as a proxy to adjust for socioeconomic status (Cyril et al., 2013). In addition, vital organ failures were associated with nutritional and immunological statuses, such as diabetes, hyperlipidemia, stroke, and hepatitis. We included these factors for analysis. This statistical methodology also enabled observational study to simulate randomized control trials.

## Discussion

This study revealed that statins were associated with a lower incidence of hepatic, renal, and heart failure but not respiratory failure. These results applied to the ACO cohort, regardless of age, sex, comorbidities, or ICS or OS use, in the late course of the study (Zeki et al., 2013; Tse et al., 2014). Past statin use may attenuate system inflammations, (Malik and Kashyap, 2003; Schierwagen et al., 2017) such as sepsis (TNF-α and IL-6) or acute lung injury (Bajwa et al., 2012; Wang et al., 2016), with multiple organ failure as demonstrated in a previous study supporting our result (Jones, 2006). Statins may be beneficial in the case of acute hepatic failure, renal failure, (Gupta et al., 2007) and heart failure. Statins significantly reduced the development of sepsis and infection-related organ dysfunction in older patients in hospitals; however, it did not reduce intensive care unit (ICU) admission incidence with respiratory failure, supporting our findings (Gui et al., 2017).

The major concerns regarding adverse statin reaction are hepatic function impairment (Jones, 2006) in chronic liver diseases and renal function impairment (Kovesdy et al., 2007). Another concern is statin use with acute lung injuries (Huang et al., 2013). Jason et al. conducted a meta-analysis of randomized trials and revealed that patients rarely discontinued statins in the event of an adverse reaction when using a high dose (Josan et al., 2008). In addition, the benefits of statins outweighed the adverse reaction in a recent report by Collins et al. (2016); this effect holds even when combined with other cholesterol-reducing drugs, such as niacin, as reported by Landry et al. (Group et al., 2014).

The lungs, heart, kidneys, and liver interact. These multiple cross-reactions make vital organ failure more complex (Vaz Fragoso et al., 2018). Those with severe forms of ACO usually received a higher frequency of ICS and OS. ICS did not present a lower risk of renal or heart failure among statin users with ACO. The ACO cohort may develop heart failure, as we noted in our previous report. Chin et al. indicated that statin therapy conferred no particular benefits to patients with heart failure undergoing percutaneous coronary intervention (Chin et al., 2018). If the ACO–statins status was steroid-resistant, ICS would not exhibit additive effects for attenuating the risk of heart failure. However, patients with ACO using both statins and ICS had the lowest risk of hepatic failure, and patients with ACO using statins and OS had the lowest risk of hepatic, renal, and heart failure. These findings are explained by statins’ additive effects on the anti-inflammatory properties of ICS and OS (Maneechotesuwan et al., 2010; Maneechotesuwan et al., 2013).

Our study also discovered that the ACO cohort had higher risks of respiratory failure and no response to statins. One explanation is that the ACO cohort may be associated with malnutrition–inflammation complex syndrome (Levin et al., 2007). Thus, hyperlipidemia and obesity protect against the development of respiratory failure. The higher body mass index and normal albuminuria in the ACO cohort indicated a lower risk of mortality than the COPD cohort. This hypothesis was supported by Bai et al. (Bai et al., 2017). Therefore, statin use in the ACO cohort did not affect the risk of respiratory failure. However, this speculation warrants further research.

The risks of hepatic, renal, and heart failure were lower among the ACO–statins status cohort. Furthermore, ICS and OS formula may attenuate the risk of hepatic, renal, and heart failure. In agreement with our result, Tsai et al. discovered that the ICS formula was associated with the lowest frequency of ICU admission based on the NHIRD (Tsai et al., 2017). Statin use in the early course of ACO may attenuate these risks in the later course of this disease.

With statins, the reported rate of adverse events differs widely, between 1–2% in randomized clinical trials (RCTs) and 10–20% in observation studies (real world). One possible explanation is the claim that RCTs mostly use a run-in period with a statin. This may exclude intolerant patients from remaining in the trial. Thus, the result may have a bias towards lower rates of intolerance. A study by Vonbank et al. includes data from RCTs with >1,000 participants with and without a run-in period who were included in the Cholesterol Treatment Trialists Collaboration. They found: 1) a majority of RCTs without a test dose of a statin in the run-in phase and 2) a test dose in the run-in phase without association of a significantly improved adherence rate within that trial comparison of the trials without a test dose. In summary, the RCTs of statins reviewed here suggest a bias towards an artificially higher adherence rate because of a run-in period with a test dose of the statin. In the review study of Vonbank et al., the apparent disparity between RCTs and observation studies are also included, albeit mostly not supported by scientific data (Vonbank et al., 2018).

Taking these together, the objective treatment of multiple organ failure should be based on the right action (e.g., blocking the inflammation-antibiotic or anti-tuberculosis drug with adjunctive therapy with a statin or steroid) at the right time (e.g., early in the course of ACO with ICS and statin before multiple vital organ failure) (Kruger and Venkatesh, 2014) in the right patients (e.g., early detection of pneumonia or pulmonary tuberculosis individualized per patient—precise medicine) (Tzovaras et al., 2011; De Loecker and Preiser, 2012; Kruger and Venkatesh, 2014; Yeh et al., 2018; Yeh, 2019). However, it should be underlined that these data come mainly from observational retrospective investigations, and randomized prospective studies are warranted to confirm these encouraging results.

## Strengths

The confirmation of the ACO cohort was established in a previous study (Yeh et al., 2018). The time-dependent model accurately presents the prescription status and correctly classifies the event-free person-time of the users before their first prescription as the unexposed follow-up time. The time-dependent model was the most informative (Levesque et al., 2010). The major cause of multiple organ failure is sepsis. In Taiwan, diagnoses of sepsis-induced vital organ failure were validated by Shin et al. (Shih et al., 2017). In addition, among Taiwan’s older population utilizing ambulatory medical services, the prevalence of certificated catastrophic illness such as hepatic, renal, heart, and respiratory failure was high. Statins were administered in accordance with Taiwanese regulations. Therefore, the diagnosis and treatment of these vital organ failures were methodical (Nan-Ping et al., 2013).

The statin effect on cardiovascular diseases based on the NHIRD has been well addressed. These studies were incorporated into the 2017 Taiwan lipid guidelines for high-risk patients (Chung et al., 2017; Li et al., 2017). The database of these previous studies is the same as in our study. Therefore, our result can provide baseline trends useful for further research on statin effect on vital organ failure (Hsieh et al., 2017; Byrne et al., 2019; Ko et al., 2019).

## Limitations

The identification of ACO challenged the researchers. PFT may increase the sensitivity to COPD (Ho et al., 2018). However, the diagnosis of ACO was based on clinical manifestation, imaging, PFT, and therapeutic response to the aforementioned bronchodilators—ICS and OS. Lanos et al. discovered that 27.1% of patients with ACO received PFT (Llanos et al., 2018). Shantakumar et al. found that 21.1% of patients with ACO received PFT after the index date in Taiwan. These laboratory data were unavailable in the NHIRD.

In our previous study, we performed propensity score matching to avoid baseline bias in the analysis of the effect of the statin on the vital organ failure (e.g., heart, hepatic, and renal failure–related diseases) such as cardiovascular diseases, pneumonia, and pulmonary tuberculosis. In this re-analysis, we found that the statin has a protective effect on cardiovascular disease, pneumonia, and pulmonary tuberculosis. Similar to these findings, the ACO cohort with statin use has a lower risk of vital organ failure, in line with our previous finings. Beta-blockers may play a role in heart failure, as in our previous study. Meanwhile, beta-blockers have an effect on heart rate control and may have a benefit with hemodynamic and clinical outcomes in patients having sepsis with respiratory failure (Coppola et al., 2015; Yeh et al., 2017). Owing to the ACO cohort with an asthma component, beta-blockers may not have an optimal role in this cohort. In addition, the ACO cohort has the experience of more severity of multiple organ failure than the non-ACO cohort (Chung et al., 2015). The drug effect on the development of multiple vital organ failure in the late course of this cohort may be more complex. These points of the study warrant further randomized control trials. Furthermore, there will be many new drugs and new strategies in the future. These points were the limitations of this retrospective study. Finally, details of the laboratory data, clinical procedure, or medical treatment records to approve the definition of ACO and organ failure were unavailable in the NHIRD. This is another limitation of our study.

In summary, the results of this study may be different in a different cohort with a different treatment strategy. However, we used a time-dependent population-based study and propensity method in this study (Yeh et al., 2019). The propensity method revealed the ACO–statin use cohort having a lower risk of hepatic (aHR = 0.51), renal (aHR = 0.45), and heart failure (aHR = 0.46) also. These methods may avoid bias in this retrospective study. Thus, our findings may contribute to the trends in the optimal use of statins in personalized medicine as part of the future of precise medicine (Godman et al., 2014; Hsieh et al., 2017; Li et al., 2017; Byrne et al., 2019; Hsieh et al., 2019).

## Conclusion

The ACO cohort using statins exhibited a lower risk of hepatic, renal, and heart failure than any other cohort, regardless of age, sex, comorbidities, or ICS or OS use of patients. The risk of hepatic failure was lower for the combined use of statins and ICS, and the risk of hepatic, renal, and heart failure was less frequent for the combined use of statins and OSs.

## Data Availability

The datasets for this manuscript are not publicly available because the dataset used in this study is held by the Taiwan Ministry of Health and Welfare (MOHW). The Ministry of Health and Welfare must approve our application to access this data. Any researcher interested in accessing this dataset can submit an application form to the Ministry of Health and Welfare requesting access. Please contact the staff of MOHW (Email: stcarolwu@mohw.gov.tw) for further assistance. Taiwan Ministry of Health and Welfare Address: No.488, Sec. 6, Zhongxiao E. Rd., Nangang Dist., Taipei City 115, Taiwan (R.O.C.). Phone: +886-2-8590-6848. All relevant data are within the paper. Requests to access the datasets should be directed to please contact the staff of MOHW (Email: stcarolwu@mohw.gov.tw) for further assistance. Taiwan Ministry of Health and Welfare Address: No.488, Sec. 6, Zhongxiao E. Rd., Nangang Dist., Taipei City 115, Taiwan (R.O.C.). Phone: +886-2-8590-6848. All relevant data are within the paper.

## Ethics Statement

The Research Ethics Committee of China Medical University and Hospital in Taiwan approved the study (CMUH104-REC2-115-CR3).

## Author contributions

The authors’ individual contributions are mentioned as follows. Conception and design: J-JY and C-HK. Administrative support: C-HK. Data collection and organization: all authors. Data analysis and interpretation: all authors. Manuscript writing: all authors. Final approval of the manuscript: all authors.

## Funding

This work was supported by grants from the Taiwan Ministry of Health and Welfare Clinical Trial Center (MOHW108-TDU-B-212-133004); China Medical University Hospital (CMU106-ASIA-12, CMU107-ASIA-19, DMR-108-207); Academia Sinica Stroke Biosignature Project (BM10701010021); MOST Clinical Trial Consortium for Stroke (MOST 107-2321-B-039 -004-); Tseng-Lien Lin Foundation, Taichung, Taiwan; and Katsuzo and Kiyo Aoshima Memorial Funds, Japan. The funders had no role in the study design, data collection and analysis, decision to publish, or preparation of the manuscript. No additional external funding was received for this study.

## Conflict of Interest Statement

The authors declare that the research was conducted in the absence of any commercial or financial relationships that could be construed as a potential conflict of interest.

## Abbreviation

ACO, asthma–chronic obstructive pulmonary disease overlap; ICS, inhaled corticosteroid; OS, oral steroid; aHR, adjusted hazard ratio; CI, confidence interval; COPD, chronic obstructive pulmonary disease.
