# Supplementary material for: Statin use and Vital Organ Failure in Patients With Asthma–Chronic Obstructive Pulmonary Disease Overlap: A Time-Dependent Population-Based Study
Source: Front Pharmacol. 2019 Aug 16;10:889. doi: 10.3389/fphar.2019.00889 (PMC6707404; doi:10.3389/fphar.2019.00889)
Supplement: Supplementary file 1 [file Table_1.docx]

Supplement Table 1. Distribution of demographic and clinical comorbidity data in study cohorts.

|  | **ACO** | | | | | |  |
| --- | --- | --- | --- | --- | --- | --- | --- |
|  | **Statin** | | | | | |  |
|  | **All(N=2392)** | | **No(N=1196)** | | **Yes(N=1196)** | |  |
| **Variables** | **n** | **%** | **n** | **%** | **n** | **%** | **p-value** |
| **Age, years** |  |  |  |  |  |  | 0.01 |
| <50 | 374 | 15.6 | 200 | 16.7 | 174 | 14.6 |  |
| 50-64 | 831 | 34.7 | 382 | 31.9 | 449 | 37.5 |  |
| 65+ | 1187 | 49.6 | 614 | 51.3 | 573 | 47.9 |  |
| Mean(SD) ^a^ | 63.4 | 12.6 | 63.8 | 13.3 | 63.1 | 11.8 | 0.17 |
| **Gender** |  |  |  |  |  |  | 0.57 |
| Women | 1256 | 52.5 | 635 | 53.1 | 621 | 51.9 |  |
| Men | 1136 | 47.5 | 561 | 48.9 | 575 | 48.1 |  |
| **Comorbidity** |  |  |  |  |  |  |  |
| Sleep disorder | 1229 | 51.4 | 624 | 52.2 | 605 | 50.6 | 0.44 |
| Diabetes | 710 | 29.7 | 361 | 30.2 | 349 | 29.2 | 0.59 |
| Hypertension | 1942 | 81.2 | 977 | 81.7 | 965 | 80.9 | 0.53 |
| Hyperlipidemia | 1829 | 76.5 | 917 | 76.7 | 912 | 76.3 | 0.81 |
| CAD | 1298 | 54.3 | 652 | 54.5 | 646 | 54.0 | 0.81 |
| Stroke | 403 | 16.9 | 196 | 16.4 | 207 | 17.3 | 0.55 |
| Hepatitis B | 40 | 1.67 | 21 | 1.76 | 19 | 1.59 | 0.75 |
| Hepatitis C | 88 | 3.68 | 43 | 3.60 | 45 | 3.76 | 0.83 |
| **Medication** |  |  |  |  |  |  |  |
| Inhaled corticosteroids (ICSs) | 619 | 25.9 | 309 | 25.8 | 310 | 25.9 | 0.96 |
| Oral steroids (OSs) | 1858 | 77.7 | 931 | 77.8 | 927 | 77.5 | 0.84 |

Chi-square test, ^a^t-test
