# Supplementary material for: Statin use and Vital Organ Failure in Patients With Asthma–Chronic Obstructive Pulmonary Disease Overlap: A Time-Dependent Population-Based Study
Source: Front Pharmacol. 2019 Aug 16;10:889. doi: 10.3389/fphar.2019.00889 (PMC6707404; doi:10.3389/fphar.2019.00889)
Supplement: Supplementary file 2 [file Table_2.docx]

Supplement Table 2. Overall incidence of hepatic failure, renal failure, respiratory failure, and heart failure (per 1000 person–years) and estimated HRs in ACO patients taking statins compared with ACO patients without statins using a time-dependent regression model by propensity scores matched.

|  | **Statin** | |
| --- | --- | --- |
| **Variables** | **No(N=1196)** | **Yes(N=1196)** |
| **Hepatic failure** |  |  |
| Person-years | 7993 | 9348 |
| Event, n | 138 | 82 |
| Rate | 17.3 | 8.77 |
| cHR (95% CI) | 1(Reference) | 0.52(0.40, 0.68)*** |
| aHR (95% CI)^a^ | 1(Reference) | 0.51(0.38, 0.66)*** |
| **Renal failure** |  |  |
| Person-years | 8297 | 71 |
| Event, n | 133 | 9528 |
| Rate | 16.0 | 7.45 |
| cHR (95% CI) | 1(Reference) | 0.46(0.35, 0.62)*** |
| aHR (95% CI)^a^ | 1(Reference) | 0.45(0.34, 0.61)*** |
| **Respiratory failure** |  |  |
| Person-years | 8825 | 9726 |
| Event, n | 4 | 3 |
| Rate | 0.45 | 0.31 |
| cHR (95% CI) | 1(Reference) | 0.69(0.15, 3.08) |
| aHR (95% CI)^a^ | 1(Reference) | 0.84(0.19, 3.85) |
| **Heart failure** |  |  |
| Person-years | 8091 | 9480 |
| Event, n | 145 | 75 |
| Rate | 17.9 | 7.91 |
| cHR (95% CI) | 1(Reference) | 0.45(0.34, 0.59)*** |
| aHR (95% CI)^a^ | 1(Reference) | 0.46(0.34, 0.61)*** |

^a^Adjusted for age; sex; comorbidity of sleep disorder, diabetes, hypertension, hyperlipidemia, CAD, stroke, hepatitis B, hepatitis C; and ICS and OS.

Abbreviations: HD, cHR: crude hazard ratio; aHR: adjusted hazard ratio; ICS: inhaled corticosteroids; OS: oral steroids.

*P < 0.05, ***P < 0.001
